# Case report: A complete lower cervical fracture dislocation without permanent neurological impairment

**DOI:** 10.1186/s12891-024-07586-9

**Published:** 2024-06-14

**Authors:** Tao Li, Xiangbin Wang, Yangmiao Ou, Yubin Long, Bin Zhu, Bei Zhao, Chaofeng Guo, Yong Li

**Affiliations:** 1 Present Address: Department of Spine Surgery and Orthopaedics, Xiangya Hospital, Central South University, Changsha, 410008 China; 2 Department of Spine Surgery, Shaoyang Central Hospital, Shaoyang, 422000 China

**Keywords:** Lower cervical spine, Fracture dislocation, Neurological impairment

## Abstract

**Background:**

Complete fractures and dislocations of the lower cervical spine are usually associated with severe spinal cord injury. However, a very small number of patients do not have severe spinal cord injury symptoms, patients with normal muscle strength or only partial nerve root symptoms, known as “lucky fracture dislocation”. The diagnosis and treatment of such patients is very difficult. Recently, we successfully treated one such patient.

**Case presentation:**

A 73-year-old male patient had multiple neck and body aches after trauma, but there was sensory movement in his limbs. However, preoperative cervical radiographs showed no significant abnormalities, and computed tomography (CT) and magnetic resonance imaging (MRI) confirmed complete fracture and dislocation of C7. Before operation, the halo frame was fixed traction, but the reduction was not successful. Finally, the fracture reduction and internal fixation were successfully performed by surgery. The postoperative pain of the patient was significantly relieved, and the sensory movement of the limbs was the same as before. Two years after surgery, the patient’s left little finger and ulnar forearm shallow sensation recovered, and the right flexion muscle strength basically returned to normal.

**Conclusion:**

This case suggests that when patients with trauma are encountered in the clinic, they should be carefully examined, and the presence of cervical fracture and dislocation should not be ignored because of the absence of neurological symptoms or mild symptoms. In addition, positioning during handling and surgery should be particularly avoided to increase the risk of paralysis.

## Background

Fracture and dislocation of the lower cervical spine is a common injury, often accompanied by different degrees of neurological impairment. Complete fractures and dislocations of the lower cervical vertebrae (in which one vertebra is completely in front of the adjacent vertebrae) are uncommon and often accompanied by severe spinal cord injury. However, in clinical practice, there are a very small number of patients with obvious cervical fracture and dislocation, but not accompanied by serious spinal cord injury symptoms, patients with normal muscle strength in the limbs or only partial nerve root symptoms, known as “lucky fracture and dislocation“ [1, 2]. We report a patient with a complete fracture and dislocation of C7 but no permanent neurological impairment. The radiological findings, treatment methods and possible injury mechanisms of the patient are discussed.

## Case presentation

**Fig. 1:**
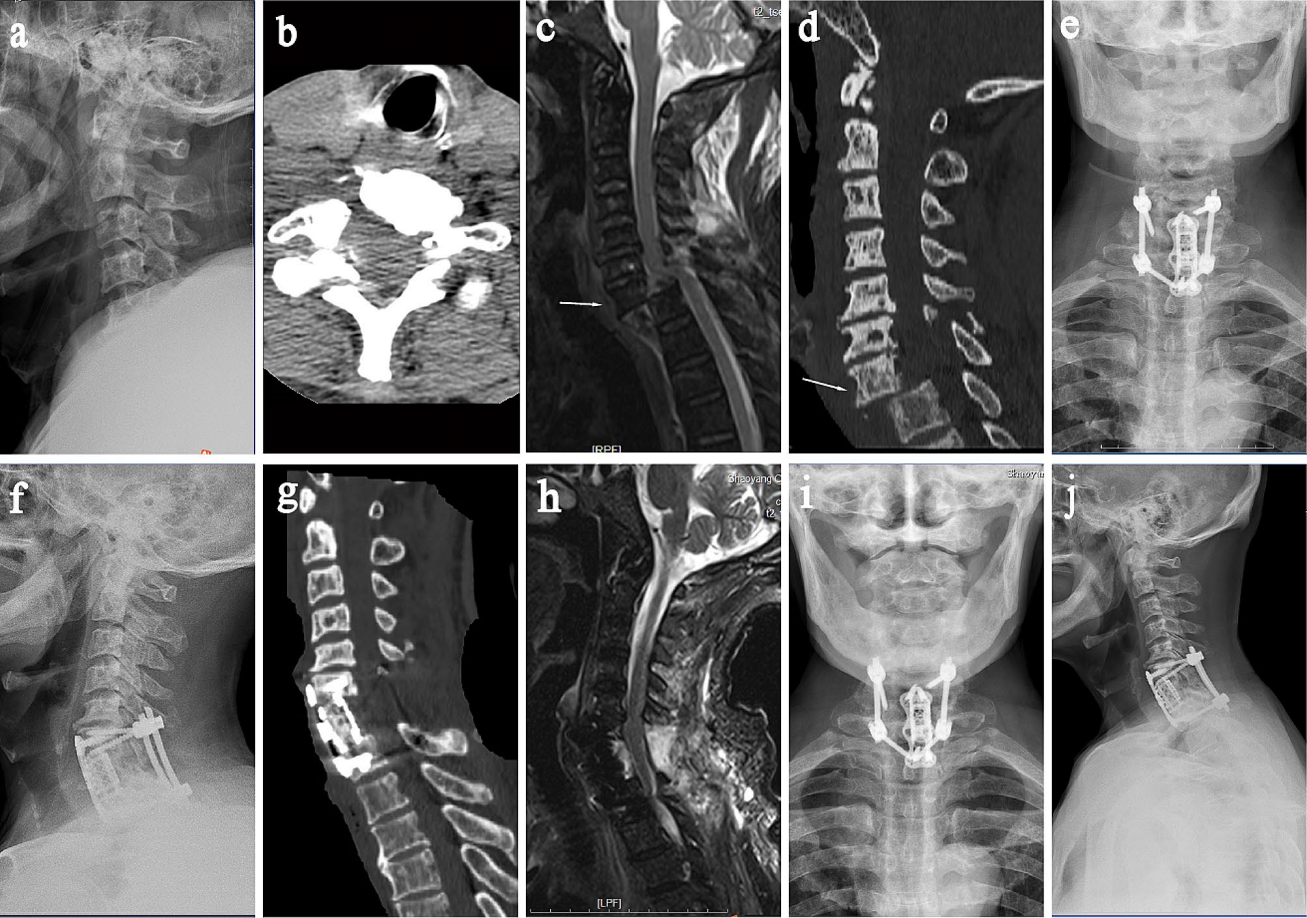
A 73-year-old male patient was admitted to the hospital with “neck pain caused by trauma for 5 hours”. (**a**) Preoperative cervical lateral X-rays showed no significant abnormalities; (**b**) Preoperative cervical CT showed that the C7 vertebral body had four degrees of anterior spondylolisthesis and C7 had bilateral adnexa and spinous process fractures; (**c**) Preoperative MRI plain scan of the cervical spine showed that the C7 vertebral body had four degrees of anterior spondylolisthesis and the cervical pulp at the corresponding level was “S” type, the C6 and C7 accessories were fractured, and the soft tissues of the neck and back were edema; (**d**) Cervical spine CT examination after traction treatment showed a slight reduction of anterior dislocation of C7; (**e, f.**) Postoperative cervical X-ray examination indicated that C6 to T1 vertebrae were in a good fixed position, and C7 vertebrae were obviously corrected for forward spondylolisthesis; (**g.**) Cervical CT examination after surgery indicated that the position of titanium cage and bone grafting were good; (**h.**) Postoperative cervical MRI showed continuity of spinal cord without obvious compression; (**i, j.**) Cervical X-ray reexamination 3 months after surgery showed the presence of physiological lordosis of the cervical spine and the position of internal fixation was good

A 73-year-old male patient accidentally fell from a height of 2 m, landing on the neck first, there were many neck and body pain, but there was sensory activity in the limbs. Physical examination: limited neck movement, tenderness in the back of the neck, decreased shallow sensation in the left little finger and ulnar forearm, grade 3 flexion muscle strength in the right hand, no obvious abnormalities in other limbs. The American Spinal Injuries Association(ASIA) grading is rated D. Preoperative cervical radiographs (day 1 after injury) showed no clear abnormality (Fig. 1a**).** Cervical computed tomography (CT) on the day of injury revealed that the C7 vertebral body had four degrees of anterior spondylolisthesis, and the C7 bilateral adnexa and spinous process fractures(Fig. 1b), and fracture of the right adnexa and spinous process of C6. Magnetic resonance imaging (MRI) examination of the cervical spine (day 1 after injury) revealed four degrees of anterior spondylolisthesis of the C7 vertebra, fractures of the C6 and 7 adnexes, and edema of the soft tissues of the neck and back (Fig. 1c). Preoperative traction with halo frame was fixed and hormone was used to reduce spinal edema. Cervical CT examination after 5 days of traction showed a slight reduction (Fig. 1d). It was discussed that posterior cervical open reduction and internal fixation were performed under general anesthesia to release the articular process strangulation, reduce and fix, then the injured intervertebral disc was removed anteriorly, the C7 vertebral body was excised, and titanium cage was placed for bone grafting. Postoperative cervical X-ray and CT examination showed that C6-T1 vertebral body was in a good fixed position, and C7 vertebral body was obviously corrected for forward spondylolisthesis (Fig. 1e-g). Postoperative MRI examination of the cervical spine showed continuity of the spinal cord without obvious compression (Fig. 1h). At discharge, the patient had no obvious pain in the neck and the whole body, no fever and dysphagia, and the sensory movement of the limbs was the same as before surgery.

Three months after surgery, the patient showed no discomfort. X-ray examination of the cervical spine showed that the cervical spine still maintained a good alignment, and no loosening or fracture of the internal fixation was observed (Fig. 1i, j). Two years after surgery, the patient’s left little finger and ulnar forearm shallow sensation recovered, and the right flexion muscle strength basically returned to normal(ASIA grading is rated E).

## Discussion

Lucky cervical fractures are rarely reported in the literature, and the most common site reported is the lower cervical vertebra (C5-C7) [2–5]. A good classification of cervical injury can help judge the degree of injury and prognosis, and also guide the choice of treatment and surgical approach. In 1982, Allen et al. proposed the Allen-Ferguson classification, which divided neck injuries into 6 categories according to the direction of injury (flexion or extension) and anatomical changes after injury (compression or separation), including compressive flexion, vertical compression, distractive flexion, compressive extension, distractive extension, and lateral flexion injury [6]. In 2007, the AO classification and Subaxial Cervical Spine Injury Classification(SLIC) and severity scale proposed by Vaccaro et al. [7] were adopted more widely. In this case, the patient should belong to the distractive flexion of Allen-Ferguson classification.

The mechanism of lucky cervical fracture is still controversial. In 1977, one such injury was reported by Pitman et al. [8]., who suggested that the external force of cervical hyperextension caused the rupture of the pedicle, resulting in “accordion-like” changes in the cervical spine. Rupture of the anterior longitudinal ligament allows the vertebral body to slide forward, the posterior structure of the fracture remains intact, and the spinal canal widens. Later, Liu et al. [1] and Tim et al. [4] suggested that this lucky fracture avoided spinal cord injury because the fracture involved the posterior structure, thus widening the spinal canal and allowing the anterior structure to shift backward, so-called “traumatic decompression of the spinal canal”. Kang et al. [9] analyzed three factors related to the degree of fracture and dislocation of lower cervical vertebra and spinal cord injury: effective space at spinal cord injury level, sagittal diameter of spinal canal at uninjured level, and Pavlov ratio at uninjured level, and concluded that the congenital width of spinal canal is the anatomical basis of severe fracture and dislocation of cervical spine without spinal cord injury. In this case, we believe that the anterior and posterior structures of the cervical spinal canal were severely damaged after trauma, resulting in enlarged anterior and posterior diameter of the spinal canal, forming a large safe space, and thus the spinal cord was displaced backward, similar to the “traumatic spinal canal decompression” described by Liu et al. and Tim et al.

Patients with lucky cervical spine fractures are easy to miss diagnosis because they do not have symptoms of paralysis, especially fractures and dislocations at C6 and C7 sites that cannot be fully developed by X-ray due to shoulder occlusion. In addition, because such patients do not have symptoms of spinal cord injury, they are often ignored and mistaken for neck soft tissue injury, delaying the diagnosis of patients. These patients have a history of significant cervical spine trauma, either flexion or extension compression injury. After injury, only neck pain, tenderness in the back of the neck, cervical spine deformity or stiffness, reduced range of motion in the neck, associated with injury segment pain and muscle spasm. In this case, X-ray could not adequately visualize C7 fracture and dislocation (Fig. 1a), while CT could diagnose it, demonstrating the value of CT scan in the early diagnosis of cervical spine fractures. Therefore, when a patient is suspected of cervical injury, the patient’s neck should be imbraked immediately, careful examination should be performed, and cervical CT and MRI should be improved as soon as possible, and the diagnosis should not be missed because of no neurological symptoms or mild symptoms.

It is precisely because such patients are not completely paralyzed that there is a risk of secondary spinal cord function impairment during future treatment, which brings great risks and challenges to treatment. The principles for the treatment of lower cervical fractures and dislocations are well established, namely early reduction, nerve decompression, and strong internal fixation [4, 10]. This type of moderate and severe injury is unstable and should be diagnosed early and treated as soon as possible. Upon diagnosis, the patient’s neck should be immobilized immediately. After neurological examination and stabilization of the general condition, cranial traction or external fixation using a Halo frame is performed to achieve stability [4]. Due to the severity of the anterior and posterior fractures, we were concerned that adding weight during traction would increase the risk of retracting the injured area, leading to iatrogenic spinal cord injury. The spinal trauma research team created the SLIC System by evaluating the injury morphology, interdisc ligament complex and nerve status, and it is believed that the score of SLIC ≥ 5 is recommended for surgical treatment, ≤ 3 is recommended for conservative treatment, and 4 is optional for surgery or conservative treatment [7]. The purpose of the surgery is to reduce and immobilize the cervical vertebra, and the internal fixation helps the patient to stabilize the fracture and reduce the risk of paralysis [11]. At present, there is controversy about the surgical method of lower cervical dislocation. Shen et al. [12] concluded that combined anterior and posterior surgery is safe and effective in the treatment of lower cervical dislocation through follow-up evaluation. The patient suffered from three-column fracture and underwent combined anterior-posterior surgery, which resulted in a high risk of intraoperative cerebrospinal fluid leakage. During the operation, we need to carefully separate the fracture block from the dura mater to reduce the incidence of cerebrospinal fluid leakage. In addition, there are complications, such as infection of the operative area, implant failure, or degeneration of adjacent segments. After operation, external fixation with braces should be strictly worn.

In this case, the patient had cervical dislocation combined with interlocked small articular process fracture, which could not be closed reduction, the SLIC score was 7, and the surgical indications were clear. The patient had injuries to all three pillars of the cervical spine, combined with a noose of the posterior articular process, which was difficult to be reduced by simple anterior surgery. In addition, the anterior pedicle of the vertebral canal was broken with small bone fragments, and simple posterior surgery may cause compression of the anterior spinal cord. Therefore, we first performed posterior open reduction, released articular process locks, reduced and fixed, then removed the injured intervertebral disc through anterior approach, excised the C7 vertebra, further decompressed the spinal cord, and placed titanium cage for bone grafting to achieve a higher fusion rate.

## Conclusion

This case report describes an elderly man who suffered a complete C7 fracture and dislocation after trauma without permanent paralysis. This case suggests that when patients with trauma are encountered, they should be carefully examined, and the presence of cervical fracture and dislocation should not be ignored because of the absence of neurological symptoms or mild symptoms. In addition, this type of cervical fracture and dislocation has been in a serious state of instability, and the positioning during handling and surgery should be particularly avoided to increase the risk of paralysis.

## Data Availability

No datasets were generated or analysed during the current study.

## Abbreviations

CT: Computed Tomography
MRI: Magnetic Resonance Imaging
SLIC: Subaxial Cervical Spine Injury Classification
ASIA: American Spinal Injuries Association
